# Evaluation of an Interprofessional Blended Learning Course Focusing on Communication within Veterinary Teams

**DOI:** 10.3390/ani14050729

**Published:** 2024-02-27

**Authors:** Sylva Agnete Charlotte Heise, Sandra Wissing, Verena Nerschbach, Ellen Preussing, Andrea Tipold, Christin Kleinsorgen

**Affiliations:** 1 E-Learning-Consulting, Centre for E-Learning, Didactics and Educational Research, University of Veterinary Medicine Hannover, 30559 Hannover, Germany; 2 Centre for E-Learning, Didactics and Educational Research, Clinical Skills Lab, University of Veterinary Medicine, 30173 Hannover, Germanyandrea.tipold@tiho-hannover.de (A.T.); 3 Clinic for Small Animals, University of Veterinary Medicine Hannover, 30559 Hannover, Germany; 4 Lightwings Preussing, VetAcademy, 8134 Adliswil, Switzerland

**Keywords:** interprofessional, team communication, teamwork, communication course, interprofessional course, blended learning, flipped classroom

## Abstract

**Simple Summary:**

This study explores the impact of a blended learning course on enhancing interprofessional communication skills among veterinary students and apprentices training to become veterinary assistants. The course, which was first implemented during the COVID-19 pandemic, utilizes a blended learning design with online modules, synchronous seminars and simulation training. The results show an increase in knowledge and improved self-assessment of communication skills, with positive feedback on the course structure. Challenges such as limited resources are noted, emphasizing the need for ongoing data collection to refine the teaching methods and maximize the benefits for participants in future runs of the course.

**Abstract:**

Based on the importance of communication and teamwork in veterinary practice, we explored the impact of a blended learning course designed to enhance interprofessional communication skills among veterinary students and apprentice assistants. The blended learning course design included online modules, synchronous (online) seminars, and simulation training sessions. The asynchronous online elements should complement the varied schedules of different professions and meet the individual needs of participants, especially considering the challenges posed by the COVID-19 pandemic. The course structure, evaluations, and outcomes were documented, showing a positive impact on knowledge gain concerning communication and self-assessment in communication skills. In the pretest, the participants scored 43.18% correct answers to a knowledge test, whereas 71.50% correct answers were given in the posttest. Some participants indicated an improvement in the self-assessment of their skills. For example, before the training only 13.64% answered the question “How prepared do you feel regarding your communication skills for entering the profession?” with “Very good” or “Good”, versus 50.00% in the posttest. There were also only 22.73% of participants who agreed to having sufficient understanding of the roles of other professional groups, while in the posttest, 81.82% agreed. The evaluations highlighted positive feedback on the organization, learning environment, and overall course structure. However, challenges such as limited resources, especially time and financial constraints, influenced the implementation and ongoing development of the course. Subsequent runs of the course could gather more data to further improve the teaching of veterinary interprofessional communication. This ongoing data collection would allow continuous insights into and adjustments to the teaching methods, ensuring maximum benefit for veterinary students and apprentice assistants.

## 1. Introduction

Communication and establishment of personal relationships between veterinarians and pet owners influence the quality and outcomes of medical treatments [1,2]. The veterinarian must actively obtain information from and provide information to the pet owner, and not just during the anamnesis. Especially after starting a treatment schedule, it is essential to maintain good communication with the owner to determine whether the animal’s health has improved or deteriorated. In the meantime, not only veterinarians and owners are often involved in the care of the animal but also other professions and groups [3]. Especially in larger practices or clinics, communication within the team is crucial to ensure the treatment is implemented and pursued efficiently and without errors. A recent study from the UK shows that not only communication with owners is important but also good team communication is crucial [4]. In this study, communication problems were identified as a contributing factor in about 80% of cases of alleged professional negligence, with about half of these including communication problems within the team or between different teams.

The successful treatment of animals often involves several specialized veterinarians, veterinary assistants, animal physiotherapists, nutrition specialists, and others [3]. This is the reason why interprofessional teamwork is gaining significance, as the success of treatments depends not only on expertise but also on effective collaboration among the professions. It is also proven that interprofessional education can positively influence the organizational climate and job satisfaction, which is shown by a scoping review based on the literature of professionals in different medical fields (except veterinary medicine), where the effect of the IPE (Interprofessional Education) of post-graduates is shown [5].

Communication skills are currently mostly taught as an elective in the veterinary curriculum in Germany [6,7,8,9,10]. A competence model including a list of communication competences has been established [11]; however, longitudinal integration and implementation are still in progress. The SOFTVETS-Competence Model [11] also includes interprofessional team communication. At the University of Veterinary Medicine Hannover (TiHo Hannover), Germany, training of communication competences for veterinary students is explicitly provided in the subject of Veterinary Professional Studies and Propaedeutics. Additional content is offered in elective courses as well as intramural practical training at the Small Animal Clinic [8]. However, the emphasis is primarily on communication with pet owners, not on team communication. In Germany’s training catalog for veterinary assistants, communication with pet owners is emphasized, but there is no explicit mention of interprofessional communication skills either [12]. Furthermore, the training of veterinary assistants differs markedly from that of veterinary students. Most of the three-year training period is workplace-based, with only short periods of vocational school blocks, while veterinary students spend 8 of 11 terms at the university and mainly gain practical experience in internships in the 9th and 10th terms. It is important to recognize that professional roles, qualifications and academic standards vary internationally. Due to differences in training structures and naming conventions across countries, there may be potential for misunderstanding [13]. In Germany, veterinary assistants undergo a three-year vocational training program organized by the regional veterinary chamber [12]. This comprehensive program includes theoretical modules alongside practical work at veterinary practices. Veterinary assistants support veterinarians in various tasks, such as animal treatment and examination, pre- and post-operative care, X-rays, client guidance and administrative duties.

A promising interprofessional pilot project was already carried out in the UK several years ago [14], but no similar educational projects have been reported in Germany, nor does the national ordinance include any adjustments toward communication or interprofessional competences in Germany yet [10]. Thus, we aim that this pilot project will lead to further curriculum development. In Germany, communication competences as well as interprofessional competences are already anchored in the national catalog of learning objectives in the study programs of human medicine [15] and dentistry [8,16]. This indicates that other healthcare sectors have already recognized the relevance, and we in veterinary medicine should also take this step.

Since the two professions of veterinarians and veterinary assistants are both trained at the TiHo, but only alongside each other and not together, a pilot project should improve this situation and close this gap in veterinary education. It should also address the lack of communication training within the team, which has not been covered in the academic curriculum and has only been thought between owner and veterinary assistant during training. In order to make a joint learning intervention accessible for both professions, a blended learning course was designed, i.e., a mixture of online learning and face-to-face learning [17]. At the time of the implementation of the course, there were still contact restrictions due to COVID-19, so face-to-face teaching was mainly reserved for the clinical–practical subjects and the elective courses mostly took place online. Therefore, the external circumstances influenced the course planning toward a blended learning concept. The course structure also simplified the organization with different professions. In order to take advantage of the benefits of the flipped classroom design, the theoretical content was initially elaborated independently in order to use the later attendance time more effectively for application and transfer tasks [18,19,20]. An elective interprofessional communication course was created and evaluated with the aim of exploring knowledge, attitudes and self-assessment of communication skills.

The research questions were as follows:

- How do participants score on a knowledge test before and after an interprofessional communication course?
- How do participants rate their self-assessment of communication skills before and after an interprofessional communication course?
- How do participants evaluate a blended learning interprofessional communication course?

## 2. Materials and Methods

### 2.1. Creation of the Course

**Figure 1:**
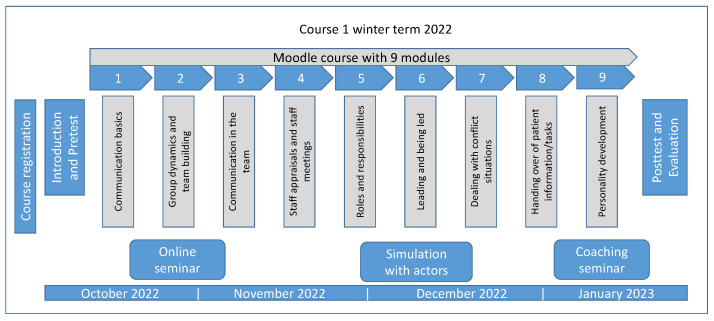
Overview of the blended learning course design from the winter term 2022/2023.

In the winter term of 2022/2023, the course “Interprofessional communication for veterinary practice” was offered for the first time to students of veterinary medicine and apprentices training to become veterinary assistants, in the following referred to as apprentice assistants. An overview of the course design is illustrated in Figure 1. We chose the concept of a flipped classroom to enable asynchronous learning of the theory to be able to use the in-person meetings more effectively to apply what has been learned [19,21,22]. This format enables personalized learning according to one’s own learning style [23]. There were no significant differences in the evaluation of the teaching approach between students of different performance levels, which indicates that all students can benefit from this concept. The basic structure of an asynchronous online course was already proven suitable for imparting communication skills [24]. It was created and provided by the TiHo Moodle platform. The course comprised nine interactive modules in which theoretical content was delivered by means of texts, videos, and interactive tasks. The tools of the Moodle platform as well as the H5P software (Versions 1.17.2) were used for this purpose. A blended learning event was chosen because online delivery of theoretical content was easily achievable and, for both professions, flexible in terms of time [25,26]. The module topics were created in accordance with the core competencies of the WHO and the recommendations of the IpEC [27,28]. Each module was available for completion for 1–3 weeks, with an average estimated processing time of 2 h. At the end of each module, tasks had to be completed to verify successful participation. These tasks included quizzes, interactive videos, audio recordings, or text composition. For the course design, thematically relevant videos were created with professional actors as best-practice or worst-case examples, focusing on typical situations and conflicts within veterinary practice.

In addition to the online course, a synchronous online seminar was conducted to delve deeper into the learning content and explore topics related to biases, potential prejudices, and professional roles. Furthermore, simulation training sessions with professional actors were offered (two hours per participant) to apply the learned theoretical content in a practical context. This training took place in the communication facility at the Clinical Skills Lab at TiHo Hannover, which is a consultation room with cameras and microphones and a one-way mirror [29]. For this purpose, six scenarios were developed, each with three versions, to encompass all the possible combinations (actor, veterinary assistant, veterinary assistant; actor, veterinary assistant, veterinarian; actor, veterinarian, veterinarian). The simulation training was designed to provide an opportunity to practice communication strategies in a safe learning environment without putting the well-being of animals or people at risk. The scenarios were debriefed through multi-source feedback (360-degree feedback) immediately following the training. The feedback was provided by participating and observing participants (veterinary student, apprentice assistant) as well as by the actors and course facilitators. Finally, one professional communication coaching event was provided, focusing on topics such as stress management and resilience.

### 2.2. Creation of the Questionnaire

To assess knowledge and attitudes regarding communication skills before and after the course, a pretest and posttest as well as an evaluation to rate the course design were developed. The individual components of the tests are listed in Table 1. To allow for a pseudonymous comparison of the results of the two tests, participants were asked to provide a unique code and some personal data. Participation was anonymized, with only the IP address allowing inferences about the identity of the participants.

The questions for the needs assessment were the original 19 items of the validated German version of the Readiness for Interprofessional Learning Scale [30]; however, the results of this analysis are not included in this paper, as these are described in more detail in another report [31]. The rating questions were designed with a five-point response scale (1 = strongly agree to 5 = strongly disagree; 1 = yes, definitely to 5 = no, not at all, 1 = very good to 5 = very poor). Self-assessment questions were also created with a five-point response scale (1 = very good to 5 = very poor, 1 = yes, definitely to 5 = no, not at all). In addition to these scales, the option “no answer” was enabled for all the questions.

The self-assessment questions and statements were based on the CSAS (Communication Skills Attitude Scale) [32] and were supplemented and validated within the working group. Similar statements have already been used in a previous study exploring veterinary students’ and apprentice assistants’ need for communication training [33].

The knowledge test section comprised 26 single-choice questions aligned with the course modules. Each single-best answer question comprised one attractor and two to four distractors. For the knowledge questions, the response “Don’t know” was also provided. An example question from this section is the following: “In which order are the teambuilding phases by Tuckman described?”.

The posttest was created similarly to the pretest. It included the collection of gender, year of birth, profession, and the unique code. The knowledge test, self-assessment, and needs assessment were repeated. In addition, a final evaluation of the event was conducted, where aspects like organization and technology, learning environment and support, sustainability and the structure of individual components were rated. The evaluation was divided into 10 different sections, consisting of a total of 66 single-choice or rating questions. Participants always had the option to select “No response”, leading to varying dataset sizes in some cases. Within these categories, 32 statements were provided for respondents to answer as rating questions. The scaling of the responses was five-point (1 = strongly agree to 5 = strongly disagree). The clarity of the nine modules was also assessed (1 = very clear to 5 = not clear) as well as the content depth (1 = too detailed to 5 = too superficial). Lastly, participants were asked to rate the helpfulness of the course modules and components (asynchronous Moodle course, synchronous online seminar, communication coaching, simulation session) for their learning success (1 = very helpful to 5 = not helpful). Finally, a filter question was asked to ascertain whether the respondent was a veterinary student or an apprentice assistant to determine the most appropriate placement of the course in their academic or training year (veterinary students: 1st to 6th academic year; apprentice assistants: 1st to 3rd training year).

For the data analysis, Microsoft Office Excel 2010 (Microsoft Corporation, Redmond, WA, USA) was used, and for the statistical analyses, SAS Enterprise Guide 7.1 (SAS Institute Inc., Cary, NC, USA) was employed.

The conducted surveys adhered to the regulations stipulated concerning general data protection. Prior to answering the questionnaires, written consent was obtained from all the participants. Additionally, a unique code was assigned to each questionnaire to ensure the collection of anonymized, matched samples. Participation was voluntary and anonymous. Apart from the raw IP addresses, no identifiable data were gathered, ensuring respondent anonymity. Participants had the option to skip any question or select “No answer”. This research adhered to the ethical standards of the University of Veterinary Medicine Hannover, Foundation. Approval for the entire project was granted by the university’s Data Protection Officer. Students who participated voluntarily provided consent for their data to be processed in line with the EU General Data Protection Regulation of 2018 (General Data Protection Regulation Art. 6 I 1 lit. e i.V.m. 89 and Lower Saxony Data Protection Act § 3 I 1 No. 1 NHG, § 13). All the collected data were analyzed and handled anonymously in accordance with the university’s data protection regulations. The university’s doctoral thesis committee, acting as the ethics committee, reviewed and sanctioned the project in line with ethical guidelines governing research involving human participants.

Upon request, the corresponding author can provide the data used in this study. However, these data are not publicly accessible due to ethical considerations and privacy restrictions.

## 3. Results

The course was offered for the first time in the winter term 22/23. Apprentice assistants (veterinary assistants in training) were informed via email, while veterinary students could enroll in the course as an elective course via online registration. In the pilot course, 18 veterinary students and 13 prospective veterinary assistants registered for the elective course. Of the latter, only 11 actually started the course and 6 of them successfully completed the entire course and additionally participated in the posttest as well. All the veterinary students completed the course in full, including assignments and tests. Altogether, 22 paired samples were collected using individual codes. All the subsequent analyses refer to n = 22, compromising 6 paired tests from apprentice assistants and 16 from veterinary students.

In the following, we will summarize the evaluations, considering “Strongly agree” and “Agree” as positive and “Disagree” and “Strongly disagree” as negative responses.

### 3.1. Demographic Data

Most of the participants were female (n = 21, 95.45%) and were born between 1984 and 2003. Less than half of them had already participated in previous communication training (n = 8, 36.36%). Almost all the participants had experience of teamwork at work (n = 21, 95.45%). Almost an equal number had already gained experience in teamwork related to sports or hobbies (n = 20, 90.91%).

### 3.2. Knowledge Test

**Figure 2:**
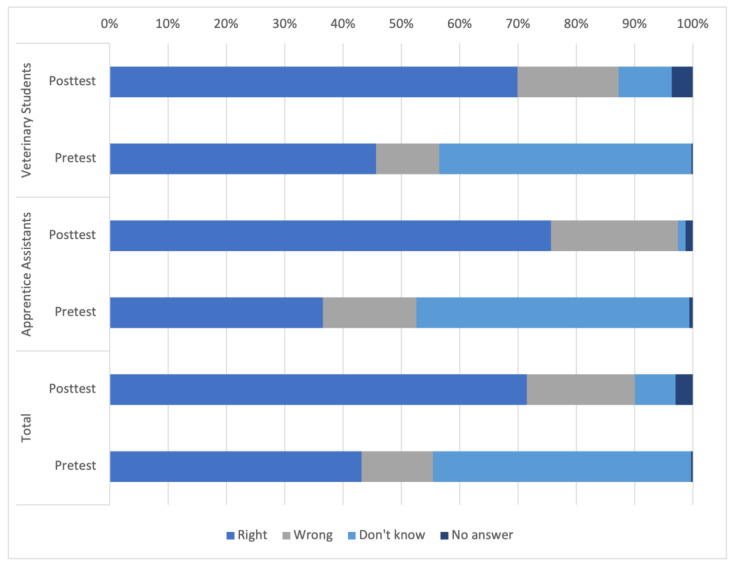
The 26 knowledge-based questions summarized and sorted by professions, presented as an overview, comparing the pre- and posttest.

In the pretest, 190 correct answers were identified in the knowledge test, while in the posttest, the number increased to 291, resulting in a knowledge gain of 53.16%. The number of incorrect answers also increased from 45 to 72, representing an increase of 60%. In the pretest, the “Don’t know” option was selected 180 times, whereas in the posttest, it was only chosen 38 times, indicating a reduction of −78.89%. Since the knowledge test questions were not set as mandatory, there was the option to continue the questionnaire without providing an answer. This was observed more frequently in the posttest than in the pretest. The differences in answering the entire knowledge test are depicted in Figure 2.

### 3.3. Communication Skills Assessment

**Figure 3:**
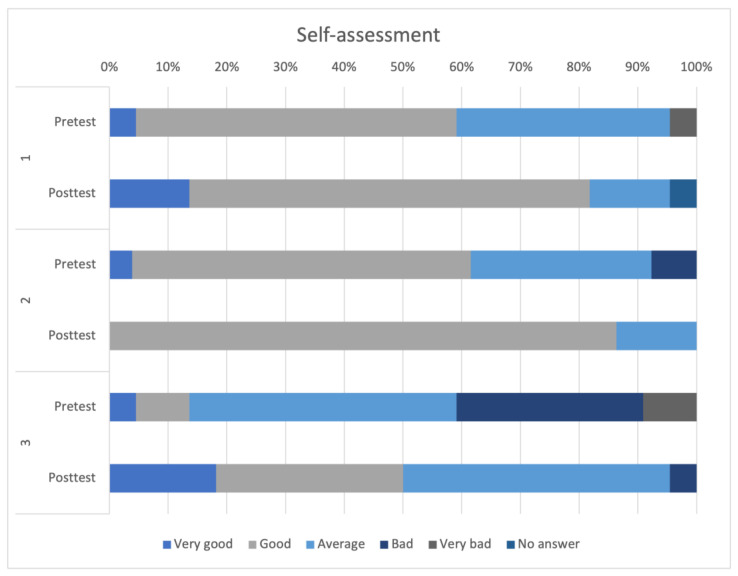
Comparative representation of self-assessment questions 1–3 between the pre- and posttest.

All three questions regarding the self-assessment of one’s communications skills showed a positive trend between the pretest and the posttest. The ratings of the three questions are shown in Figure 3. The most obvious change was observed in question three. The response “Very good” was chosen more than three times as often in the posttest (n = 4, 18.18%) compared to the pretest (n = 1, 4.55%), and the same applied to “good” (Pretest: n = 2, 9.09%, Posttest: n = 7, 31.82%). In the posttest “Very bad” was no longer selected and the selection of “Bad” decreased to approximately one-sixth of its previous frequency (Pretest: n = 7, 31.82%, Posttest: n = 1, 4.55%).

Self-assessment questions 1–3:

1. How do you rate your communication skills with pet owners?
2. How do you rate your communication skills within a team?
3. How prepared do you feel regarding your communication skills for entering the profession?

**Figure 4:**
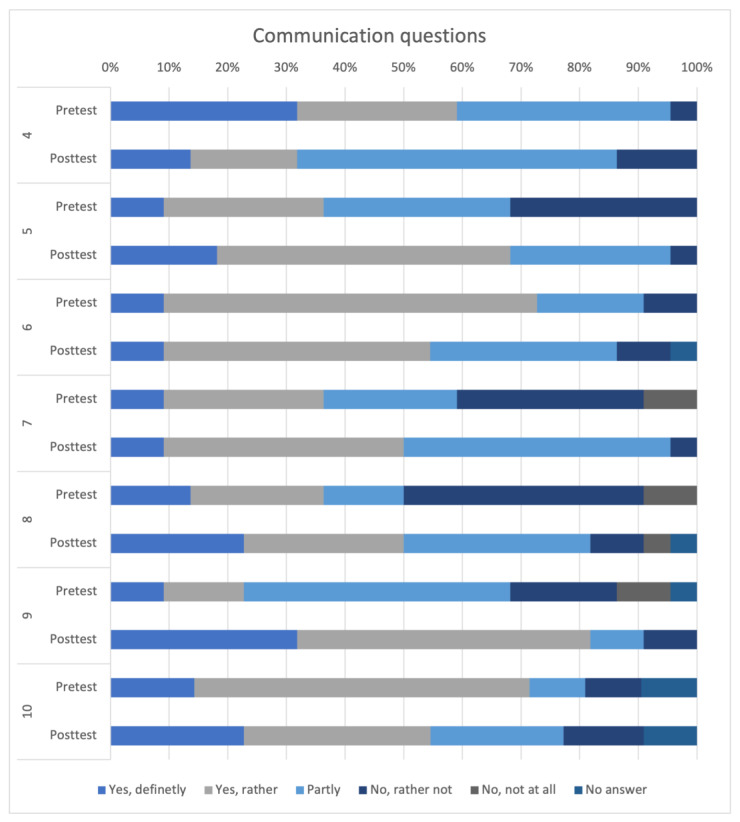
Comparative representation of self-assessment questions 4–10 between the pre- and posttest.

The additional questions regarding one’s communication skills and their assessment are shown in Figure 4. The majority of the participants saw room for improvement in their communication skills in the pretest and chose “Yes, definitely” or “Yes, rather” (n = 13, 59.09%). In the posttest, this value decreased by about half (n = 7, 31.82%). In the pretest, a huge number of participants rated themselves as not prepared for potential conflicts within the team (n = 7, 31.82%) and two participants even chose “No, not at all”. In the posttest, these selection options were almost not chosen at all.

Self-assessment questions 4–10:

4. Do you see room for improvement in your communication skills?
5. Do you feel well-prepared for employee discussions?
6. Can you delegate tasks effectively to others?
7. Do you feel well-prepared for potential conflict situations within a team?
8. Has your study/training helped you acquire communications skills?
9. Do you feel you have sufficient understanding of the roles of other professional groups?
10. Do you wish for interprofessional events in the timetable?

**Figure 5:**
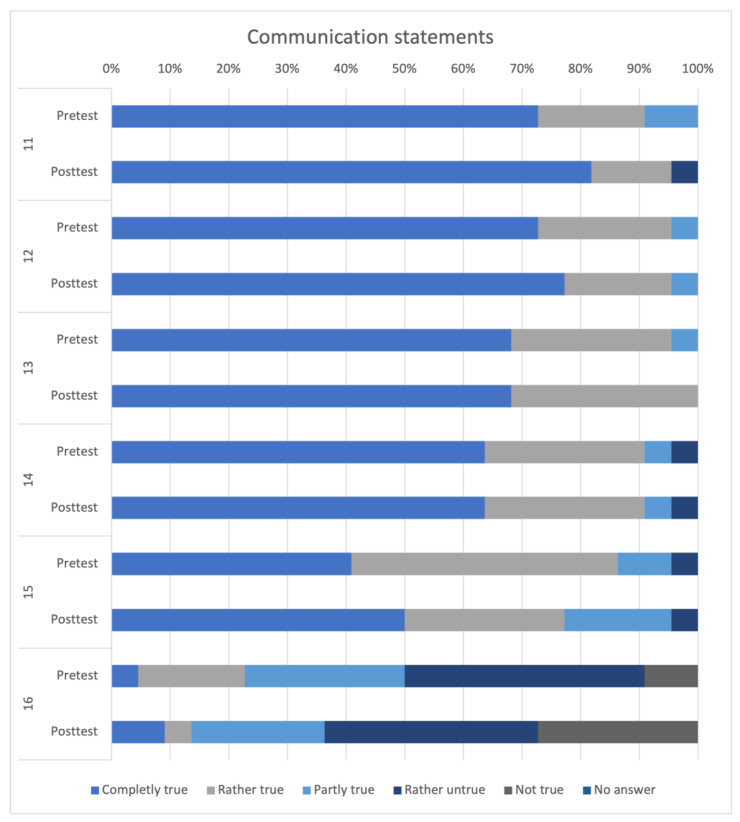
Comparative representation of self-assessment statements 11–16 between the pre- and posttest.

Communication statements 11–16 are depicted in Figure 5. With the exception of statement 16, these were all rated very positively. Statements 11 to 15 were not rated differently in the pre- and in the posttest. The statement “I don’t have time in my educations/studies to learn communication skills”. received higher ratings of “Rather not true” and “Not true” in the posttest (n = 14, 63.63%) than in the pretest (n = 11, 50%).

Communication statements 11–16:

11. Communication skills are important for my everyday life.
12. Learning communicative skills enhances my ability to conduct customer/patient conversations.
13. Learning communicative skills improves my ability to communicate within a team.
14. Through communication training in education/studies, I can learn to better handle conflict situations in my future professional life.
15. I believe that communication skills can be learned.
16. I don’t have time in my education/studies to learn communication skills.

### 3.4. Evaluation

The evaluation of the course was filled out 25 times; in the following, we only focus on 22 of the paired samples.

#### 3.4.1. Organization and Technology

The majority of the respondents found the organization of the event to be well-executed (n = 21, 95.45%). Participation in the Moodle course also went smoothly for the majority (n = 19, 86.36%). The overall course concept, which included an asynchronous online course, (online) seminars, and in-person meetings, received positive ratings (n = 20, 90.91%).

#### 3.4.2. Learning Atmosphere and Support

**Figure 6:**
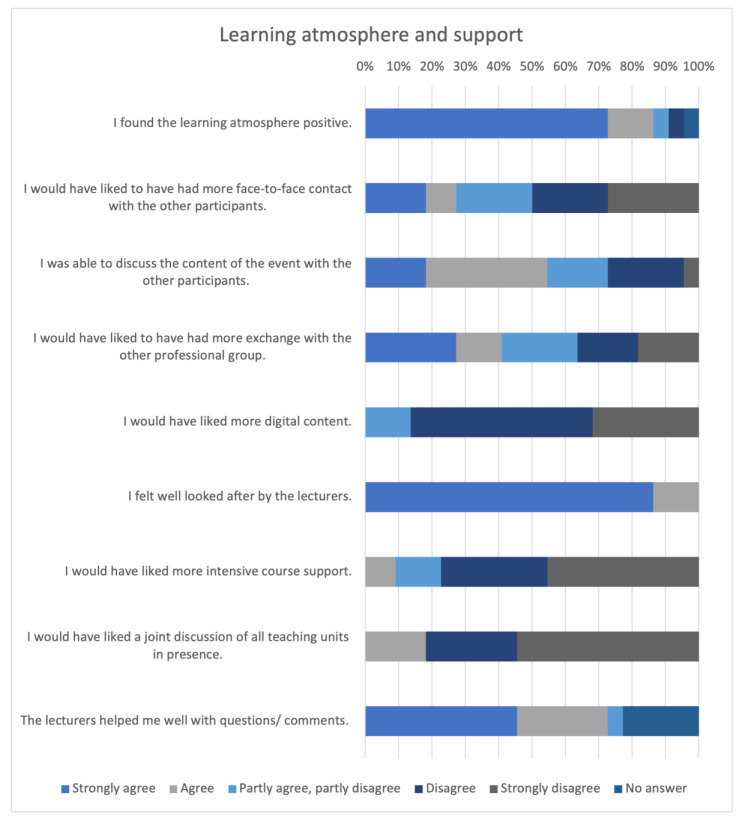
Evaluation of the learning atmosphere and the support.

The results of the evaluation part “Learning atmosphere and support” are shown in Figure 6.

Most participants evaluated the learning environment positively (n = 16, 72.73%, “Strongly agree”; n = 3, 13.64%, Agree”). In response to the statement “I would have liked more face-to-face contact with other participants”, varied responses arose. Nonetheless, more participants disagreed with this statement (n = 5, 22.73% “Disagree”, n = 6, 27.27% “Strongly disagree”).

Participants indicated that they had sufficiently compared notes with other participants about the course content (n = 18.18%, n = 4 “Strongly agree”; n = 8, 36.36% “Agree”). No participant chose to agree with the item “I would have liked more digital content”. All the participants felt well looked after by the lecturers. Five participants chose not to select an answer for the item “The lecturers helped me well with questions/comments”.

#### 3.4.3. Sustainability

**Figure 7:**
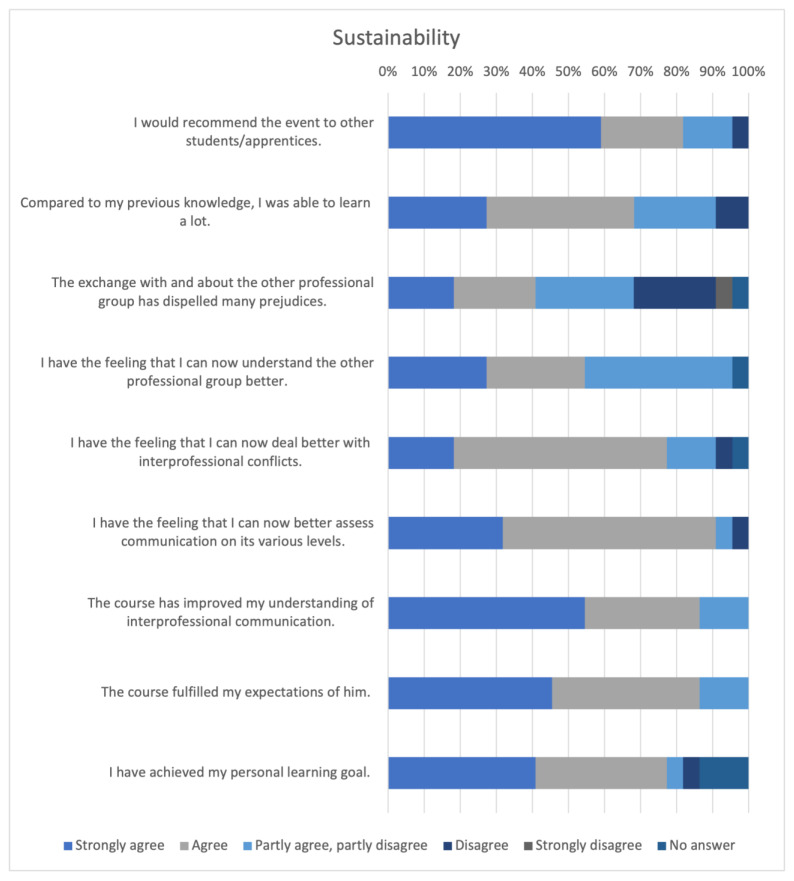
Evaluation of the sustainability.

The results from the section “Sustainability” of the evaluation are shown in Figure 7.

Most participants stated that they would recommend the event to other veterinary students/apprentice assistants (n = 18, 82.81%). Regarding the extent of the learning achieved, most participants agreed that they had learned a lot (n = 15, 68.18%). The respondents stated that their understanding of the other respective professional group had improved (n = 12, 54.55%). The course mostly fulfilled the expectations of the participants (n = 19, 86.36%).

#### 3.4.4. Structure

**Figure 8:**
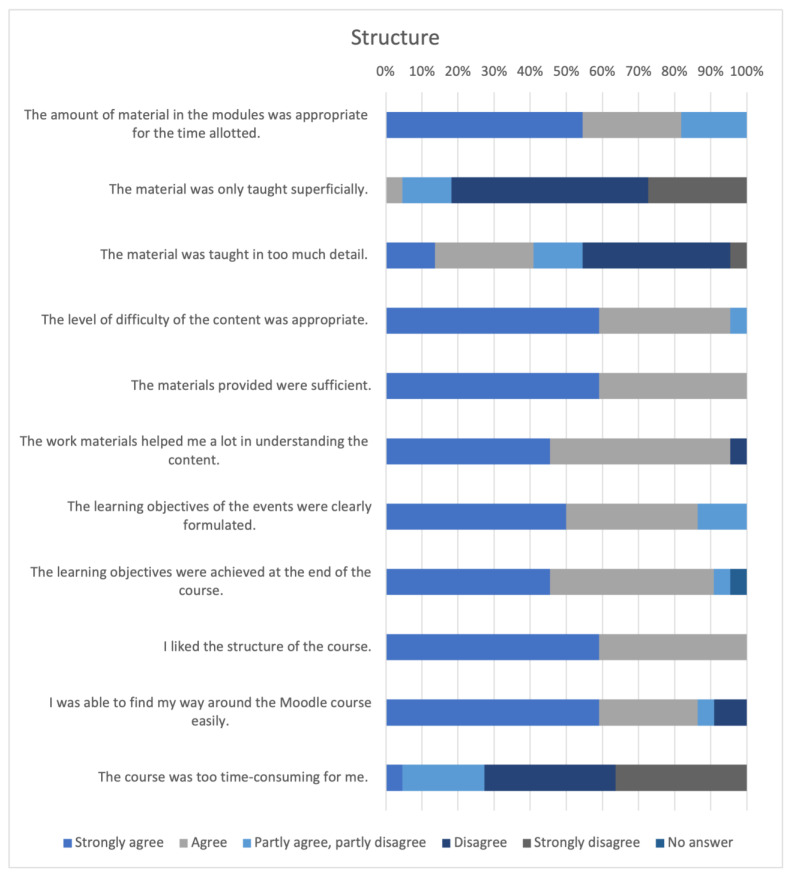
Structure evaluation.

All the results of the section “structure” of the evaluation are displayed in Figure 8.

The majority of participants rated the amount of content as appropriate for the scheduled period (n = 18, 81.82%). Most respondents disagreed that the content had been covered too superficially (n = 18, 81.82%). The level of difficulty was mostly rated appropriate (n = 21, 95.45%). All the provided learning materials were rated as sufficient (n = 22, 100%). All the participants liked the structure of the course (n = 22, 100%).

#### 3.4.5. Comprehensibility

Six of the nine modules were exclusively rated as “Very understandable” and “Rather understandable”. In the modules “Communication basics” and “Leading and being led” and “Personal development”, one person each gave “Partly” as an answer for these modules. Additionally, in the “Leading and being led” module, one person also marked it as “Rather incomprehensible”.

#### 3.4.6. Professional Depth

The professional depth of all the online course modules was rated by the participants on a scale from “Much too detailed” to “Much too superficial”. All the modules were mostly rated as “Exactly right”.

**Figure 9:**
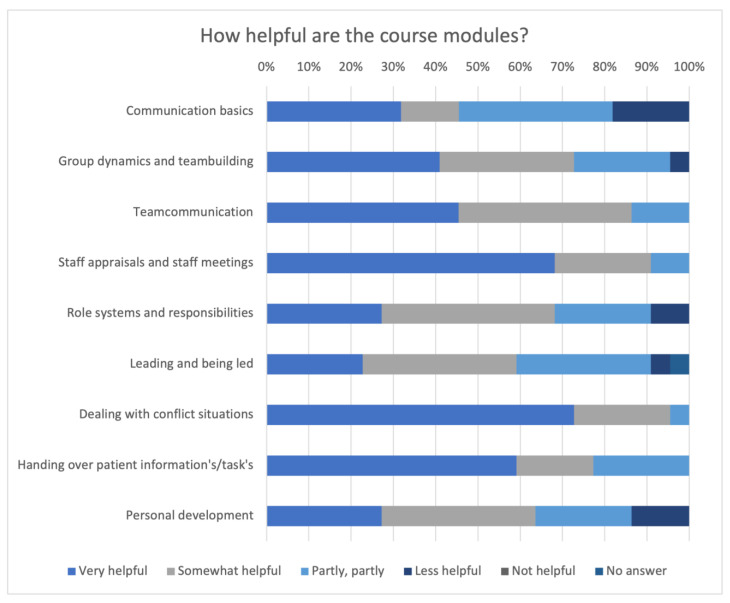
Presentation of the evaluation of the rating of the helpfulness of the modules.

All the results of the evaluation of the helpfulness of the modules are shown in Figure 9. The module “Staff appraisals and staff meeting” was the best rated module (n = 20, 90.91%). The module “Communication basics” was the lowest rated module (n = 12, 54.55%).

**Figure 10:**
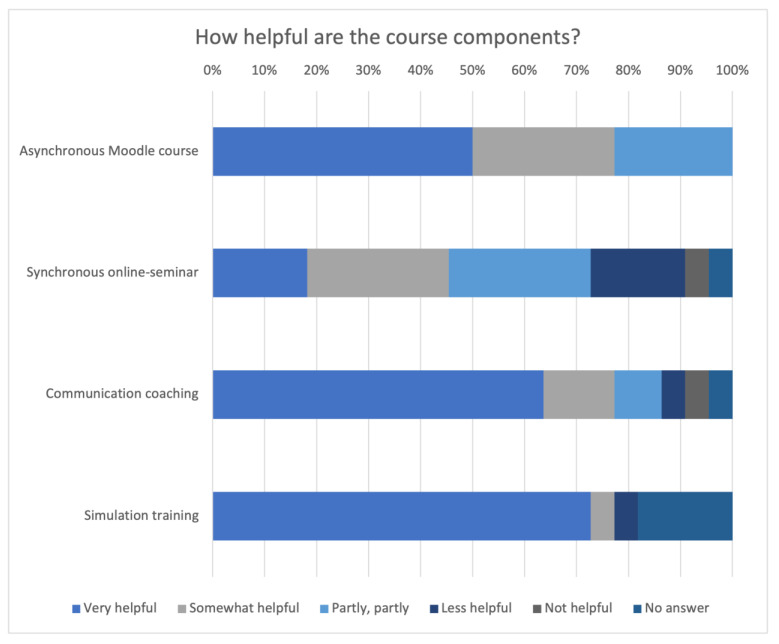
Evaluation of the rating regarding how helpful the components of the course are.

Figure 10 presents all the results of the rating of the components’ helpfulness. The simulation training was the best rated part of the course (n = 17, 77.27%), whereas the lowest rated component was the online seminar (n = 5, 22.73%).

The veterinary students and apprentice assistants were asked in which academic or trainee year they would find an interprofessional communication course appropriate. The veterinary students were in strong agreement, particularly recommending the third academic year (n = 11, 68.75%). The apprentice assistants’ responses were less conclusive, with half of the respondents favoring the first year of training (n = 3, 50%).

## 4. Discussion

Communication is unanimously recognized as a core competency in the medical and veterinary health professions [34,35]. Although this competency has not yet been mandatorily incorporated into veterinary medicine, it is taught to varying extents at German universities [8,10]. As part of the interprofessional communication course in the winter term 22/23, a positive effect on the knowledge and self-assessment of the communication skills of veterinary students and apprentice assistants was observed. Furthermore, the evaluation confirmed that both professional groups positively evaluated the blended learning course, particularly the simulation training. The implementation and reporting of any course evaluation on interprofessional courses in the veterinary field are relatively rare [14]. Even if there are papers about communication courses in this field [36,37,38] and about interprofessional courses in other healthcare sectors [39,40,41], the gap in veterinary medicine is huge.

The pilot project “Interprofessional Team Communication for Veterinary Practice” enjoyed a positive response, which is an indication to us that veterinary students and apprentice assistants appreciate the relevance of the subject matter. Several studies also show that the attitude toward interprofessional education is mostly positive, even if a lot of students do not have much knowledge about it [42,43]. Our aim was to demonstrate the potential of an interprofessional learning intervention in veterinary medicine. After the initial veterinary interprofessional intervention in England in 2011 yielded positive results [14], the anticipated curriculum adjustments did not materialize. Therefore, more than 10 years later, with this project in Germany, we aimed to take the next step in this direction and focus on the course structure to help to close the research gap. With this project, we also sent a response to the various calls for action from overarching organizations [28,44]. The alternative to interprofessional learning is uniprofessional education, which is the current format being used mostly in veterinary medicine, especially for veterinary students.

Implementing the course went smoothly for the veterinary students, whereas the apprentice assistants encountered greater difficulty in completing the course, as measured by the number of apprentice assistants who did not complete the course entirely. That could be due to the fact that veterinary students are required to fulfill a specific number of elective hours, whereas the apprentice assistants voluntarily took the course alongside their regular workload in the clinics and received only a certificate of attendance for it. Improvement is needed in this regard. Time resources need to be provided to potential participants to successfully engage in interprofessional courses with similar prerequisites and motivations. Previous studies also indicate that it is not only the apprentice assistants who may have encountered difficulties in completing the course, even students seemed to prefer the blended learning approach; they also mentioned lack of discipline and problems with their time management as being obstacles preventing them from completing blended learning courses [45]. These might also be reasons especially contributing to the issues faced by the apprentice assistants.

The improved self-assessment of the participants’ communication skills post-course provides an indication of the effectiveness of the multi-week training, even if studies show that the self-assessment is not always an accurate tool to measure clinical competencies, especially for low-performing students [46]. A study indicated that also shorter durations resulted in fewer measurable differences among participants but showed positive changes on the part of the pet owners [47]. As already assessed by Bahramsoltani et al. [37], especially the practical training is useful for an improvement in communications skills. This was also obvious in our evaluation when comparing the online course with the practical learning sessions. The blended learning concept also facilitated the organization of the course with the different professions and different daily routines. Nevertheless, the evaluation indicated a desire for more interaction. In particular, the simulation training was resource-intensive. In addition to preparing the scenarios and training and paying the actors, time and space capacities were also limitations.

An extensive evaluation was administered as a posttest, directly after completing the course. The course structure proved suitable for the event, although some participants wished for more in-person interaction during the course. As aforementioned, asynchronous online courses were already proven suitable for imparting communication skills in veterinary education [24]. The asynchronous elements combined several advantages, like flexibility, repeatability, and better understanding through multimedia use [48]. Combined with the practical training, we used the already established flipped classroom concept [22,49]. Blended learning is an already approved learning method for veterinary medicine, especially for clinical skills like communication [50]. Furthermore, there are also hints that the learning outcome after blended learning compared to traditional learning is higher [21,26]. However, every teaching and learning model’s natural has its advantages and disadvantages. This format allows students to tailor their learning to their individual needs. The course design was chosen because unnecessary contacts should still be avoided at TiHo Hannover due to COVID-19 infection numbers [51]. The use of videos to reflect on simulated situations is particularly suitable for training the ability to perceive and reflect [52]. After completing the pilot course, our aim was to continue and to make the content available and accessible for further persons and professions. Due to the limited resources, the course was exclusively conducted online, with two synchronous online seminars covering the topics prejudices and conflict potentials and resilience and stress management. Also, in the next winter term, the course was offered again. This time complemented by two additional online modules covering the topics of the seminars, as synchronous online seminars posed a challenge in finding an available time slot for all the professions. There is particular scope here to create opportunities for online collaboration and teamwork in asynchronous settings. More feedback and especially peer-centered activities were reported by students who studied mostly in synchronous settings in comparison with mostly asynchronous settings [53]. Furthermore, it must be highlighted how virtual collaboration is defined and to what extent virtual collaboration is achievable within asynchronous settings [54]. Also, the need for technical and facilitating support for online group processes and tasks with shared purposes must be considered in course design and, in particular, during the implementation.

The long-term aim of this pilot project is the implementation of an interprofessional communication course, or ideally, the longitudinal integration of this content into the curriculum. In this context, the major hurdles in the medical field have already been explored regarding implementation, with organizational challenges being prominent and highly qualified instructors playing a crucial role in the execution [55]. There was previously also a study that measured the benefit of team communication training, the results of which showed an improvement in productive teamwork after such training [56]. It is shown that especially with the longitudinal implementation, further challenges arise in logically embedding individual content into various sessions [57]. However, resources are also described as a limiting factor. Faculties that have not yet offered integrated interprofessional teaching mainly cite funding limitations and limited participation from other disciplines as reasons [58]. Both arguments we can understand well after piloting the course and running a second and third adapted learning intervention with limited financial resources.

It should be emphasized that although the apprentice assistants performed slightly lower on average in the pretest regarding the knowledge-based questions (36.54% correct answers) compared to the veterinary students (45.67%), they performed even better in the posttest (75.64% correct answers by apprentice assistants, 69.95% correct answers by veterinary students). Regarding the knowledge test, as was to be expected, an increase in knowledge from 53,16% could be demonstrated by the increase in the number of correct answers after completion of the course. However, there is still room for improvements as not all participants were able to answer all the questions correctly. Therefore, improvements could either be made in the knowledge test or also in teaching and learning material to achieve better outcomes. The chosen answer “Don’t know” decreased, which also speaks in favor of the increase in competence. Nevertheless, we also observed an increase in incorrect answers in the posttest. This observation shows an increase in self-confidence in choosing a wrong answer rather than selecting “Don’t know” and can be described as a supposed increase in competence. The guessing of answers, instead of choosing “Don’t know”, could be observed in the Progress Test of Veterinary Medicine as well [59]. This is a phenomenon that has not yet been fully explained by studies, but also in the Progress Test of Veterinary Medicine and a study about self-regulated learning the amount of both correct and incorrect answers increased with growing knowledge, while the “Don’t know” response decreased [59,60]. These findings suggest a notable boost in self-assurance, implying that the uptick in incorrect responses can also be construed as a positive advancement. However, it has been already shown that veterinary medical students do not always assess their own abilities realistically [46].

Due to the small sample size resulting from a small number of participants, particularly the even smaller number of paired samples, the study’s statements are limited. We had a relatively small sample size, further skewed by the disproportional distribution of the two surveyed professions. While the sample size of this study may not initially appear particularly large, the response rate of the apprentice assistants was very high. Out of a total of 21 apprentice assistants working at the University of Veterinary Medicine, 13 enrolled in the course. Furthermore, generating connected samples proved to be challenging, as some codes could not be assigned to the participants. Similar issues have been observed in other studies at the TiHo [61]. Because of the sample number, we focused on descriptive analysis in this paper.

The pretest was conducted immediately before the course began, and the posttest was administered immediately afterwards. To assess the sustainable learning outcomes and long-term changes in attitude and self-assessment, it would be beneficial to conduct additional tests at intervals of several weeks to months after the completion of the course, as in a previous study [62]. We used the same questions in the pre- and posttest, but the test bias is likely to be minimal in our study, as several months elapsed between the two tests and neither the questions nor the correct answers were disclosed. As a further outlook, we anticipate running additional iterations of the course after the pilot phase. These iterations can provide us with comparative insights into which teaching format is most effective. By attaining a larger sample size, this would enable us to explore differences among various professions.

## 5. Conclusions

Overall, the blended learning approach received positive feedback, proving to be organizationally beneficial and suitable for our event. Looking ahead to the continuation of the course, we anticipate further evaluations that will likely yield additional insights. Bearing in mind that communication teaching in veterinary medicine still receives little attention, with this positive example of an interprofessional course, we hope to influence curriculum adjustments and demonstrate the relevance and benefits of interprofessional education.

Additional investigation is warranted to explore the relationship between learning, shifts in performance or attitudes and the resulting impact on healthcare outcomes. This area of inquiry remains open for further exploration, with the potential to deepen our understanding of the mechanisms underlying educational interventions in healthcare settings and their ultimate effect on patient care and job satisfaction. Based on our findings, we can encourage other institutions to incorporate interprofessional education. Furthermore, there is a need for communication training, which is currently not adequately offered to all. Incorporating simulation training in various contexts, including interprofessional settings, could help improve communication in future workplaces. Collaboration among different educational institutions is necessary to provide access to such courses for various professions. To mitigate organizational challenges, the blended learning concept appears to be a promising approach for the implementation of interprofessional training in veterinary and veterinary associated professions.

## Figures and Tables

**Table 1:** Overview of the pre- and posttest questions.

	Number of Questions	
Question Type	Pretest	Posttest
Demographic data	8	3
Knowledge test	26	26
Needs assessment (RIPLS) [30]	19	19
Self-assessment	16	16
Previous experience	1	0
Course expectations	4	0
Evaluation questions	0	69

## Data Availability

All the data presented in this study are available on request from the corresponding author. The data are not publicly available due to ethical and privacy restrictions.
